# Bacteriome and mycobiome dysbiosis in oral mucosal dysplasia and oral cancer

**DOI:** 10.1111/prd.12558

**Published:** 2024-03-19

**Authors:** Georgios N. Belibasakis, Chaminda Jayampath Seneviratne, Ruwan Duminda Jayasinghe, Phuc Thi‐Duy Vo, Nagihan Bostanci, Youngnim Choi

**Affiliations:** ^1^ Division of Oral Diseases, Department of Dental Medicine Karolinska Institutet Stockholm Sweden; ^2^ School of Dentistry The University of Queensland Herston Queensland Australia; ^3^ Department of Oral Medicine and Periodontology, Faculty of Dental Sciences University of Peradeniya Peradeniya Sri Lanka; ^4^ Department of Immunology and Molecular Microbiology, School of Dentistry Seoul Korea

**Keywords:** bacteriome, dysbiosis, microbiome, mycobiome, oral cancer, oral candidiasis, oral dysplasia, oral medicine, oral mucosa, oral pathology

## Abstract

It has long been considered that the oral microbiome is tightly connected to oral health and that dysbiotic changes can be detrimental to the occurrence and progression of dysplastic oral mucosal lesions or oral cancer. Improved understanding of the concepts of microbial dysbiosis together with advances in high‐throughput molecular sequencing of these pathologies have charted in greater microbiological detail the nature of their clinical state. This review discusses the bacteriome and mycobiome associated with oral mucosal lesions, oral candidiasis, and oral squamous cell carcinoma, aiming to delineate the information available to date in pursuit of advancing diagnostic and prognostic utilities for oral medicine.

## 1 THE ORAL MICROBIAL ECOLOGY

The oral cavity, encompassing both mucosal and hard dental surfaces, is colonized by an amplitude of microorganisms collectively defined as the oral microbiota. This includes bacteria, fungi, viruses, and archaea. They tend to live in harmony with the host in a symbiotic state. Yet, changes in the composition of the normally dominant taxa shift the composition of the regional microbial community in favor of a disease, which is often coupled with the term “dysbiosis.”^1^ To decipher these microbial community shifts, we need to understand that they are tightly connected to the net ecological conditions and nutritional availability of the colonized site or niche. The concept of “biogeography” implies that distinct microanatomical sites are selective for different microbial communities, tightly dependent on the prevailing ecological conditions.^2^, ^3^ Oral microbial communities therefore tend to be niche specific from a qualitative (number of taxa) or a quantitative (abundances of taxa) perspective,^1^, ^4^ and their metabolic products may modify the surrounding microenvironment, shaping further the microecological queues within.^2^, ^5^

## 2 THE ORAL MUCOSA AND THE EFFECT OF MICROBIAL DYSBIOSIS

The oral mucosa is an important barrier that protects the oral cavity from various external influences, including mechanical trauma, temperature extremes, chemicals, and pathogens. In addition, it plays a vital role in host immunity by fostering immune cells across its tissue layers, helping to recognize and respond to exogenous pathogens or foreign substances. From childbirth, the oral mucosa is naturally colonized by oral microbiota, establishing a critical dynamic for maintaining a state of “symbiosis” or “eubiosis.” This interaction is constantly influenced by lifestyles and environmental factors such as diet, smoking, and consumption of tobacco products, which can alter the microbiome composition and properties, thus causing imbalance and eventually “dysbiosis,” compromising the integrity of the oral mucosa. A myriad of pathologies may cause the loss of structural and functional integrity of the oral mucosa. These can be broadly categorized into four groups: neoplasms (benign, precancerous, and malignant), infectious diseases (bacterial, fungal, and viral), autoimmune disorders, and trauma.^6^ The foregoing pathologies invariably affect the microbiota residing on the oral mucosa. While dysbiosis of the mucosal microbiome may not always result in an apparent clinical infection, it can well exacerbate existing pathologies or lead to secondary ones. Moreover, continuous release of microbial toxins and the resulting induction of chronic inflammation may contribute to mutagenesis, epithelial dysplasia, and tissue damage.

## 3 ORAL SQUAMOUS CELL CARCINOMA AND DYSBIOSIS

Oral squamous cell carcinoma (OSCC) represents the most prevalent form of malignant neoplasm affecting the oral cavity, with a global annual incidence of approximately half‐million new cases per year.^7^ Despite advancements in surgical interventions, adjuvant radiotherapy, and chemotherapy, the overall 5‐year survival rate for OSCC patients remains at approximately 50%–60%.^8^ Cigarette smoking, alcohol consumption, tobacco chewing, and genetics are widely recognized as major risk factors for the development of OSCC.^9^ In recent years, there has been increasing interest in the role of the oral microbiome in the development of OSCC.

Since Virchow's observations in 1863, the concept of the interplay among “bacteria, inflammation, and cancer” has been proposed,^10^ although the notion that oral infections could play a role in cancer was generally dismissed prior to 1980s.^11^ Around 1994, the involvement of bacterial infection in the development or progression of cancer was demonstrated by the association of *Helicobacter pylori* with gastric cancer, leading to the classification of *H. pylori* as a human carcinogen by the World Health Organization's International Agency for Research on Cancer. The presence of *H. pylori* has also been reported in dental plaque and saliva.^12^ However, only few studies examined the presence of *H. pylori* in OSCC, with rather conflicting findings. Some suggested a correlation between *H. pylori* presence and OSCC progression, while others did not find significant association or even reported a protective effect.^13^, ^14^

**FIGURE 1:**
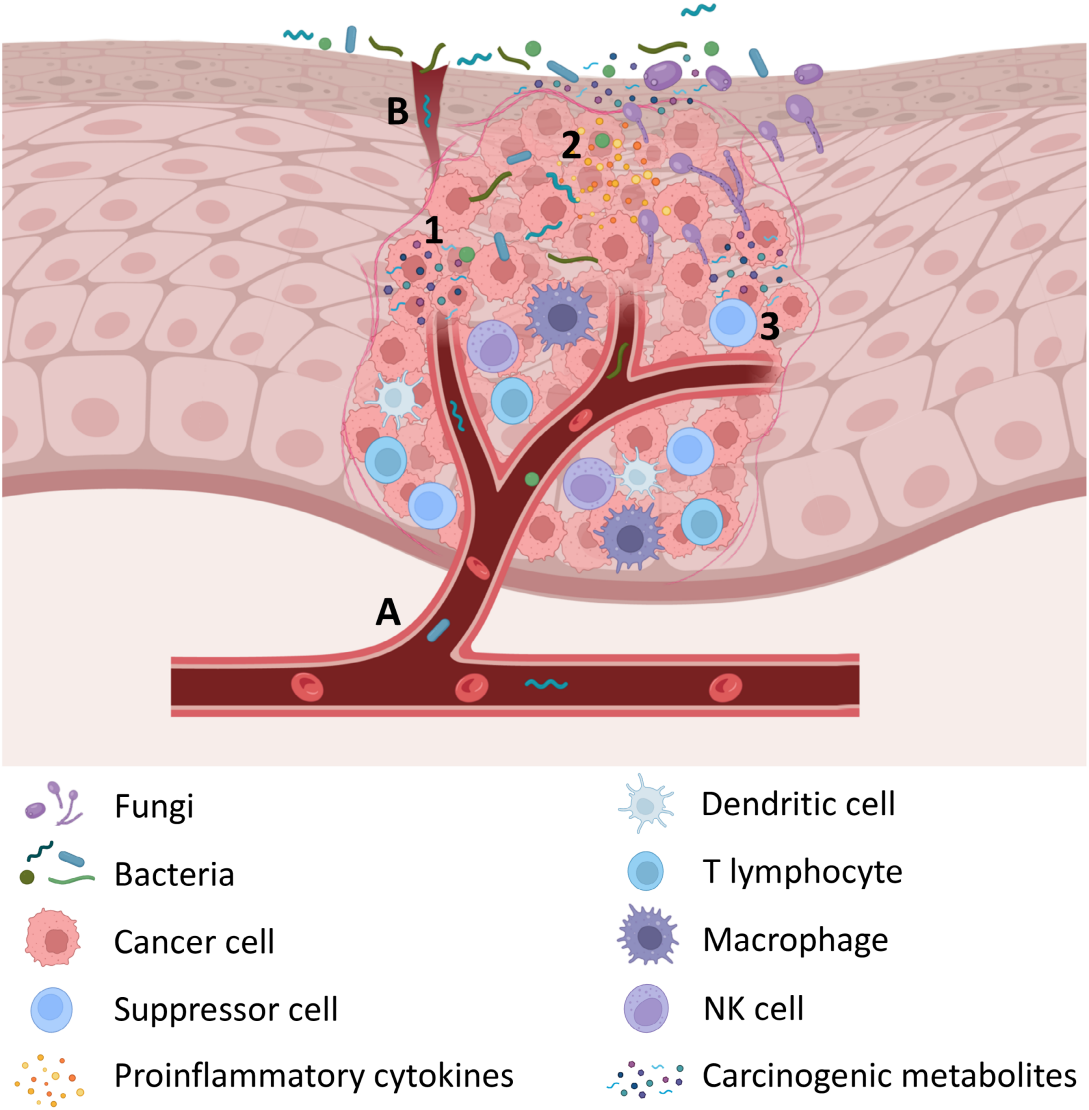
Potential contribution of bacteria and fungi on the development of OSCC. Bacteria may enter the bloodstream (A) or translocate from the periodontal pocket or microinjury (B) to the tumoral site. Proposed contributing mechanisms of bacteria and fungi to the development of OSCC include the production of carcinogenic metabolites (1), induction of proinflammatory microenvironment (2), and alteration of the balance between host cell proliferation and apoptosis (3). Figure was generated and adapted from “Tumor Microenvironment 2,” by BioRender.com (2023), retrieved from https://app.biorender.com/biorender‐templates.

A bacterial role in carcinogenesis may encompass the alteration of the microenvironment towards the establishment of a proinflammatory milieu where the production of carcinogenic metabolites is prominent (Figure 1).^15^ It is reasonable to question whether alterations in the vast and diverse composition of the oral microbiome, or persistence of oral bacterial infection, could constitute a catalytic cause of OSCC. Notably, modifications in the microbial community are frequently observed in common oral diseases, such as periodontitis and caries, driven by polymicrobial dysbiosis and overgrowth of pathobionts. Periodontal pathobionts such as *Porphyromonas gingivalis* have been associated with oral premalignant lesions and OSCC.^16^ This may be partly due to the endogenous nature of oral infections and the potential regulatory influence of oral bacteria on inflammation. The dysbiotic microbiome within the tumor microenvironment likely contributes to tumor progression by sustaining chronic inflammation. Hence, the following sections will provide insights into the OSCC‐associated microbial communities found in various sample sources, including oral rinses, saliva, plaque, tumor swabs, biopsy sections, and tissue homogenates. Tumor swabs and homogenates provide evidence of bacterial colonization or penetration of the tumor site. In contrast, biopsy sections stained with immunohistochemistry can reveal intracellular localization of bacteria.

## 4 ORAL TUMOR SURFACE‐ASSOCIATED BIOFILMS

Earlier association studies between microbial profiles and OSCC have focused on swab analysis. Reportedly. Nagy et al. were the first to identify compositional differences in the microbial community colonizing the surface of OSCC versus the adjacent clinically healthy tissue. They performed culture‐based analysis of OSCC surface swabs and found that the collected biofilms harbor significantly increased numbers of oral aerobes and anaerobes, as compared to healthy mucosal surfaces of the same patient.^17^ The same study also showed that tumor sites harbored increased numbers of *Veillonella*, *Fusobacterium*, *Prevotella*, *Porphyromonas*, *Actinomyces*, *Clostridium*, *Haemophilus*, *Enterobacteriaceae* and *Streptococcus* spp. *Candida albicans* was present in approximately one‐third of the tumor sites, but never in healthy sites. Schmidt et al. analyzed swabs from the lesion surface and contra‐lateral normal mucosa obtained from OSCC patients, precancer cases, and healthy controls. They isolated DNA from the swab samples and applied 16S amplification and 454 pyrosequencing to profile the obtained microbial biofilm. The abundance of *Firmicutes* (e.g., *Streptococcus*) and *Actinobacteria* (e.g., *Rothia*) was found to be significantly decreased in cancer samples, compared to contralateral normal mucosa surface from the same patient. Significant decreases in abundance of these phyla were also observed in swabs from precancerous lesions, but not in samples from tongue and floor of mouth of healthy individuals. The OSCC samples were associated with significantly higher proportions of *Fusobacterium* spp., whereas phylum *Bacteroidetes* was notably more abundant in both cancerous and normal tissue surfaces of OSCC patients, suggesting that increased colonization by this phylum is associated with an increased risk for OSCC. In a similar work, Zhao et al. identified drastic changes in the bacterial communities of OSCC lesions by 16S rRNA sequencing on an Illumina MiSeq platform. They reported that phyla *Spirochaetes*, *Fusobacteria*, and *Bacteroidetes* were enriched at the OSCC lesions while *P. gingivalis* did not differ in abundance between the groups.^18^ Additionally, Zhang et al.^19^ reported a higher abundance of 10 bacterial species in biofilms associated with OSCC, some of which were common with those identified by Zhao et al. The shared species included *Aggregatibacter segnis*, *Capnocytophaga leadbetteri*, *Peptostreptococcus stomatis*, and *Prevotella intermedia*. They also reported an enrichment of *Campylobacter rectus*, *Catonella morbi*, *Fusobacterium nucleatum*, *Gemella morbillorum*, *Peptococcus* spp., and *Porphyromonas catoniae* at the tumor sites, and reversely an enrichment of *Granulicatella adiacens*, *Granulicatella elegans*, *Corynebacterium matruchotii*, and *Streptococcus oralis* on healthy buccal mucosa. Comparison of predicted microbial functions between the OSCC‐associated and control microbiota revealed an increased abundance of bacterial genes involved in lipopolysaccharide, co‐factor and vitamin biosynthesis, as well as bacterial chemotaxis. Hence, the distinctive composition of bacterial biofilms at the tumor site may support a potential role for the oral bacteriome in oral carcinogenesis and tumor progression. However, most members of the OSCC‐associated bacteriome were anaerobes, and it is thus conceivable that the tumor microenvironment selectively promotes the growth of specific tumor‐associated bacteria.^18^

## 5 ORAL TUMOR INTRATISSUE MICROBIOTA

Despite being an invasive procedure, tissue biopsy is the gold standard for diagnosing OSCC. The tumor microbiota within seems to develop through selection by the tumor microenvironment, shaping these diverse microbial communities. It is perceived that deeper tissues remain largely devoid of bacteria in healthy individuals, but malignant tumors tend to be more susceptible to bacterial colonization. A plausible mechanism is that bacteria can transiently enter the bloodstream and gain access to the interior of the tumor (Figure 1). This is facilitated by the disorganized or “leaky” tumor vasculature, which forms in response to pro‐angiogenic growth factors, produced by both cancer cells and the surrounding stromal cells.^20^ A potentially more persistent source would be bacterial translocation from the ulcerated periodontal pocket due to increased permeability of the junctional epithelium, or from other areas of the oral mucosa due to microinjury (Figure 1). It is worth noting that the inadequate blood supply within solid tumors often results in the formation of hypoxic areas, creating favorable conditions for the growth of anaerobes. These bacteria tend to preferentially colonize nutrient‐rich, anoxic, and necrotic tumor regions. Periodontal pathobionts, such as *P. gingivalis* and *F. nucleatum*, are enriched under such microenvironmental conditions.^19^, ^21^, ^22^, ^23^ Indeed, *F. nucleatum* can co‐aggregate with *P. gingivalis* in multi‐species biofilms and plays a crucial role in establishing a vital capnophilic environment that supports the growth of the later.^24^ Immunohistochemical analysis of paraffin‐embedded samples of OSCC and noncancerous gingival mucosa both showed positivity for *P. gingivalis*, but at significantly higher levels in OSCC.^25^ The bacterial community within OSCC tissues appears to be viable and distinct from that found in the healthy marginal tissue surrounding the tumors.^26^ This community consists of up to 52 different phylotypes,^27^ including *Streptococcus*, *Veillonella*, and *Rothia* spp.^28^, ^29^ Further on, Al‐Hebshi et al.^30^ reported as many as 228 different bacterial species in OSCC tissues with *F. nucleatum* being the most abundant one, followed by *Pseudomonas aeruginosa*, a bacterium in fact rarely encountered in the oral environment. Functional prediction analysis in OSCC samples revealed the prevalence of genes associated with bacterial mobility, flagellar assembly, bacterial chemotaxis, and lipopolysaccharide biosynthesis, revealing an enriched intra‐tumoral “inflammatory bacteriome.”^19^, ^30^, ^31^ Also, *C. albicans* is increased within the OSCC tumor, advocating for an intra‐tumoral “inflammatory mycobiome” that can act in concert with the bacteriome in facilitating carcinogenesis and tumor progression.^32^ It is also important to consider that variations in the composition of the oral microbiome of OSCC are dependent on the tumor site, or the sampling site of the tumor. For instance, the abundance of *Prevotella intermedia* was profoundly higher in the gingiva, whereas *F. nucleatum* was the most enriched in OSCCs next to the tongue.^33^

Yet, in cancer, the metabolism and functionality of the specialized bacterial community may be more relevant than its composition.^16^ While the connection between microbial community composition and OSCC has received much attention, comprehensive studies on the molecular function are lacking. In a pilot study, Yost et al.^34^ conducted a community‐wide meta‐transcriptome analysis of entire oral microbiome in OSCC sites, adjacent nontumor regions, or among healthy controls, finding that within OSCC tissues *F. nucleatum* displayed the highest biological activity. In line with these findings, *F. nucleatum* has been associated with other cancer types including pancreatic, colorectal, and esophageal.^35^, ^36^, ^37^ The ability of *F. nucleatum* to modulate the tumor immune microenvironment by enhancing its survival and capacity to recruit tumor‐infiltrating immune cells indicates that this species may accelerate tumor progression through the induction of inflammation.^38^, ^39^, ^40^ However, there is at present no consensus on whether *F. nucleatum* acts as a neoplastic “initiator” or as a “thriving survivor” within the OSCC microenvironment. There is, however, evidence that the functional inhibition of cytotoxic T‐cells and the recruitment of regulatory T‐cells create an immunosuppressive microenvironment, allowing survival of the tumor cells.^41^ Increased numbers of *F. nucleatum* have been reported in colorectal cancer as a result of an immunosuppressed microenvironment, which in turn impairs regional immune surveillance.^42^

## 6 SALIVARY DYSBIOSIS AND OSCC

The oral cavity provides a distinctive opportunity for screening individuals at risk of oral cancer. The visible nature of the lesions allows for direct observation through minimal or noninvasive sampling. Several noninvasive sampling methods have been employed for the detection and analysis of OSCC. These include collection of saliva and oral rinse, based on the assumption that the microbiota in such samples may differ, thus reflecting the differences in the composition between the normal mucosa‐associated versus tumor‐associated biofilms. In recent years, high‐throughput technologies have facilitated the widespread use of “omics” to identify biomarkers and targets for OSCC in saliva. Various microbes, metabolites, proteins, transcribed genes, miRNAs, genome alterations, and epigenomic changes have been proposed for oral cancer diagnosis.^43^, ^44^, ^45^, ^46^, ^47^, ^48^, ^49^ A recent diagnostic test called “CancerDetect,” which utilizes RNA‐sequencing analysis with 270 human and microbial mRNA features, demonstrated a specificity of 94% and a sensitivity of 90% in detecting OSCC,^50^ paving the way for salivary diagnostic tools to detect OSCC.

Several studies have reported that certain common oral bacteria are elevated in OSCC with increased colonization of facultative oral streptococci and anaerobic genera, including *Prevotella*, *Veillonella*, *Porphyromonas*, *Capnocytophaga*, *Peptostreptococcus*, and *Fusobacterium*.^51^, ^52^, ^53^ A targeted species analysis employing checkerboard DNA–DNA hybridization identified elevated levels of *Capnocytophaga gingivalis*, *Prevotella melaninogenica*, and *Streptococcus mitis* in the saliva of OSCC patients, and combinations of these species were found to reliably predict 80% of cancer cases with a specificity of 83%.^27^, ^54^ Furthermore, three anaerobes, *Prevotella tannerae*, *F. nucleatum*, and *P. intermedia*, were associated with a higher risk of OSCC in another study.^55^

## 7 SERUM ANTIBODY PROFILING IN OSCC

Profiling autoantibodies generated against tumor‐associated antigens is a promising tool for diagnosing or predicting the course of the disease or the response to therapy in OSCC.^56^, ^57^ However, only few studies have investigated the usefulness of serum antibody levels against candidate pathobionts in diagnosing this pathology. In an OSCC cohort study, elevated levels of antibodies to *P. gingivalis* were observed in OSCC patients, compared to non‐OSCC controls, although sensitivity reached 53.2% and specificity 84.4%.^58^ Similarly, in the extensive National Health and Nutrition Examination Survey III, oro‐digestive cancer mortality was found to be related to the levels of *P. gingivalis* antibodies.^59^ These findings indicate the need for inclusion of antibody arrays to identify a broader spectrum of microbial drivers of OSCC.^60^

## 8 BACTERIAL DYSBIOSIS IN ORAL POTENTIALLY MALIGNANT DISORDERS

If OSCC‐associated bacterial dysbiosis plays a role in carcinogenesis, it is expected that common patterns of oral bacteriome dysbiosis would be identified in early oral mucosal lesions with the potential to progress to OSCC. Oral potentially malignant disorders (OPMD), as defined in the 2020 WHO workshop, encompass oral leukoplakia (OL), oral erythroplakia (OE), proliferative verrucous leukoplakia (PVL), oral submucous fibrosis (OSF), oral lichen planus (OLP), actinic keratosis, palatal lesions in reverse smokers, oral lupus erythematosus, dyskeratosis congenita, oral lichenoid lesions, and chronic oral graft‐versus‐host disease.^61^ Among these, studies on the oral microbiota in OL, OE, PVL, OSF, and OLP have mainly been conducted, likely due to their high prevalence or elevated malignant transformation rates (Table 1). Studies comparing oral bacteriota in OPMD and healthy individuals or healthy sites, using target‐independent high‐throughput sequencing of the 16S rRNA gene or metagenome, were further considered. The analyzed samples included tissue biopsies, mucosal surface swabs, saliva, or oral rinse. Studies on OPMD‐associated oral bacteriota are collated under OL and OLP (Tables 2 and 3).

**TABLE 1:** Prevalence and malignant transformation rates of common oral potentially malignant disorders.

	Global pooled estimated prevalence^a^	Cumulative rate of malignant transformation^b^
Leukoplakia	4.11% (95% CI = 1.98–6.97)^a^	9.5% (99% CI = 5.9–14.0)
Erythroplakia	0.17% (95% CI = 0.07–0.32)^a^	33.1% (99% CI = 13.6–56.1)
Proliferative verrucous leukoplakia	A rare subtype of leukoplakia	49.5% (99% CI = 26.7–72.4)
Oral submucous fibrosis	4.96% (95% CI = 2.28–8.62)^a^	5.2% (99% CI = 2.9–8.0)
Oral lichen planus	1.01% (95% CI = 0.74–1.32)^b^	1.4% (99% CI = 0.9–1.9)

Abbreviation: CI, confidence interval.

^a^ Values by meta‐analysis (Mello et al. 2018,^80^ Gonzalez‐Moles et al. 2021^66^).

^b^ Values by meta‐analysis (Iocca et al. 2020^81^).

**TABLE 2:** Characteristics of microbiome studies on oral premalignant lesions.

Authors	Sample	Group	Country	Method	Statistics	α‐diversity	β‐diversity	Comments
Hu et al., 2016^82^	Saliva	10 OL 19 HC	China	16S rRNA	*p* < 0.05	↑	Different	No consideration of confounders
Amer et al., 2017^83^	Mucosal swab	36 OL (Lesion + HS) 32 HC	Ireland	16S rRNA V1‐V2	*p* < 0.05 LDA > 3.0 & *q* < 0.015	versus HC: ↓ versus HS: N.S	Different Different	Consideration of age, dentures, smoking, drinking, and oral hygiene Exclusion: diabetes mellitus, Crohn's disease, ulcerative colitis, current viral infection, history of gastrointestinal malignancy, antibiotics, or topical steroids therapy within 6 months
Hernandez et al., 2017^84^	Mucosal swab Saliva	10 PML 112 no PML	USA	16S rRNA V3‐V5	*q* < 0.05	N.S	Different	PML includes leukoplakia, erythroplakia, and submucous fibrosis Consideration of age, sex, ethnicity, betel nut chewing, smoking, drinking, HPV status, diabetes, BMI, and antibiotics therapy within 6 months
Lee et al., 2017^52^	Saliva	124 PML 127 HC	Taiwan	16S rRNA V4	*p* < 0.05	N.S	N.S	PML includes dysplasia, hyperplasia, and hyperkeratosis Consideration of age, sex, betel chewing, smoking, drinking, and family history of cancer Exclusion: diabetes mellitus, immune system‐related disease, and antibiotics therapy within 3 months Change in the salivary microbiome network is maintained from PML to OSCC
Decsi et al., 2019^85^	Biopsy	7 PML (Lesion + HS)	Hungary	Metagenomics (pooled DNA)	‐	‐	‐	PML includes verrucous hyperplasia, lichenoid gingivitis, lichenoid buccitis with hyperplasia, reactive changes, or basal cell dysplasia Consideration of age, sex, systemic disease, allergy, smoking, drinking, oral hygiene, oral sexual activity, and medication consumption Exclusion: abnormal vital signs, pregnancy, antibiotics or corticosteroids therapy, and large‐dose probiotics consumption within 2 months
Hashimoto et al., 2019^86^	Saliva	6 OL 4 HC	Japan	16S rRNA V4	*p* < 0.05	‐	‐	Consideration of dysplasia level, age, sex, medical history, smoking, and drinking Exclusion: antibiotics therapy within 3 months, current infectious disease, and history of malignant disease
Ganly et al., 2019^87^	Oral rinse	8 OL 12 HC	USA	16S rRNA V3‐V4	*q* < 0.1	N.S	N.S	OLK with dysplasia, nonsmoking, and HPV negative Consideration of age, sex, ethnicity, and drinking Adjustment for age and drinking Periodontal pathogens increased along HC‐PML‐OCSS
Gopinath et al., 2020^88^	Saliva	20 OL 23 HC	India	16S rRNA V3‐V4	sPLS‐DA	N.S	Different	OLK with moderate or severe dysplasia Consideration of age, smoking, and drinking Exclusion: co‐existing debilitating illness and antibiotics therapy within 1 month
Herreros‐Pomares et al., 2021^89^	Biopsy	10 PVL 5 HC	Spain	16S rRNA V3‐V4	*q* < 0.05	↓	Different	Consideration of dysplasia level, OSCC transformation, age, sex, and smoking

Abbreviations: HC, healthy controls; HS, healthy sites; LDA, linear discriminant analysis; N.S, not significant; OL, oral leukoplakia; OSCC, oral squamous cell carcinoma; PML, potentially malignant lesions; PVL, proliferative verrucous leukoplakia; sPLS‐DA, sparse partial least squares discriminant analysis.

**TABLE 3:** Characteristics of microbiome studies on oral lichen planus.

Authors	Sample	Group	Country	Method	Statistics	α‐diversity	β‐diversity	Comments
Choi et al., 2016^90^	Mucosal sample	13 OLP 11 HC	Korea	16S rRNA	*p* < 0.05	N.S	Different	Consideration of severity score, pain score, duration, age, sex, and systemic disease with medication Exclusion: antibiotics or steroid therapy within 1 month, xerostomia, and smoking Increase species associated with gingivitis or periodontitis
Wang et al., 2016^91^	Saliva	19 R‐OLP 18 E‐OLP 18 HC	China	16S rRNA V4	*p* < 0.05	N.S	N.S	Consideration of severity score, sites, age, sex, and BMI; no periodontal inflammation, caries, severe systemic disorders, smoking, drinking, and OLP treatment within 2 months
He et al., 2017^92^	Buccal scraping	21 E‐OLP 22 NE‐OLP 21 HC	China	16S rRNA V3	*p* < 0.05	↑	‐	Consideration of age and sex Exclusion: tumor, systemic diseases, dentures, salivary gland diseases, caries, periodontal diseases, smoking, drug abuse, antibiotics therapy within 1 month, immunomodifier within 6 months, and pregnancy or lactation
Carvalho et al., 2019^93^	Saliva	2 OLP 2 NSIL	Brazil	16S rRNA V3‐V4	*p* < 0.05	↑	‐	Consideration of age, sex, smoking, symptoms, medication, dentures, hypertension, and diabetes
Du et al., 2020^94^	Mucosal swab	10 R‐OLP 10 E‐OLP 10 HC	China	16S rRNA V3‐V4	*p* < 0.05 LDA > 3.5	↑	‐	Consideration of severity score, age, and sex Exclusion: severe systemic disease, salivary gland disease, untreated periodontal or caries lesions, smoking, OLP treatment within 3 months, antibiotics or glucocorticoids therapy within 3 months, and pregnancy or lactation
Hijazi et al., 2020^95^	Mucosal swab	18 OLP 13 HC	UK	16S rRNA V1‐V2	*q* < 0.05	↓	N.S	Consideration of severity score, pain, lesion sites, age, sex, BMI, pocket depth, Candida, and salivary flow rate Exclusion: chronic medical conditions, deranged hematological and biochemical profiles, abnormal vital signs, BMI >30 or < 20, smoking, pregnancy or lactation, antibiotics therapy within 3 months, reduced salivary flow rate, dentures, medications, therapy for oral ulcers within 3 months, other oral mucosal diseases, periodontal disease, caries, and high‐sugar diet
Wang et al., 2020^96^	Saliva	20 R‐OLP 20 E‐OLP 20 HC	China	16S rRNA V3‐V4	*p* < 0.05	N.S	N.S	Consideration of symptom score, sign score, lesion sites, age, sex, BMI, smoking, drinking, and teeth number Exclusion: other oral mucosal diseases, tumors, severe systemic disease, lichenoid reactions caused by drugs/amalgam filling, pregnancy, lactation, contraceptives, mouthwash usage within 7 days, periodontitis, caries, dentures, OLP treatment within 2 months, antibiotics therapy within 1 month, and immunomodifier drugs within 3 month
	Biopsy	12 R‐OLP 8 E‐OLP 4 HC				N.S	Different	
Deng et al., 2020^94^	Saliva	17 E‐OLP 17 NE‐OLP 17 HC	China	16S rRNA V3‐V4	*q* < 0.05	N.S	N.S	Consideration of severity score, lesion sites, age, and sex Exclusion: systemic or immunological diseases, skin lesions or other oral infectious diseases, and no relevant treatment within 3 months
Yu et al., 2020^97^	Saliva	10 E‐OLP 10 NE‐OLP 10 HC	China	16S rRNA V4	*p* < 0.05	N.S	Different	Consideration of age, sex, and saliva pH; no hypertension, diabetes, smoking, and drinking
Zhong et al., 2020^98^	Saliva	10 OLP 5 HC	USA	RNA	*p* < 0.05	N.S	N.S	Consideration of lesion sites, OLP treatment, age, sex, and comorbidity Exclusion: xerostomia, systemic or oral infection, periodontitis, plaque, antibiotics therapy, and history of cutaneous lichen planus
Zheng et al., 2021^99^	Buccal scraping	23 E‐OLP 20 NE‐OLP 48 HC	China	16S rRNA V4	*p* < 0.05	↑	Different	Consideration of lesion sites, age, and sex Exclusion: systemic diseases, tumors, salivary gland disease, menstruation, pregnancy or lactation, periodontal disease, antibiotics therapy within 1 month, and immunomodulator drugs within 6 months
Li et al., 2021^100^	Saliva	30 OLP 21 NC	China	16S rRNA V3‐V4	*p* < 0.05	↑	Different	Consideration of age, sex, and *H. pylori* infection Exclusion: periodontitis, caries, systemic disease, smoking, and drinking
Chen et al., 2022^101^	Mucosal swab	77 OLP (lesion + AS) 76 HC	China	16S rRNA V3‐V4	*q* < 0.05	versus HC: ↓ versus AS: N.S	Different N.S	Consideration of age and sex Exclusion: OLP treatment within 3 months, antibiotics or glucocorticoids therapy within 3 months, other oral mucosal disease, severe systemic diseases, pregnancy or lactation, periodontal disease, caries, and missing teeth >3

Abbreviations: AS, adjacent sites; E‐OLP, erosive OLP; HC, healthy controls; LDA, linear discriminant analysis; N.S, not significant; NE‐OLP, nonerosive OLP; NSIL, nonspecific inflammatory lesions; OLP, oral lichen planus; R‐OLP, reticular OLP.

**FIGURE 2:**
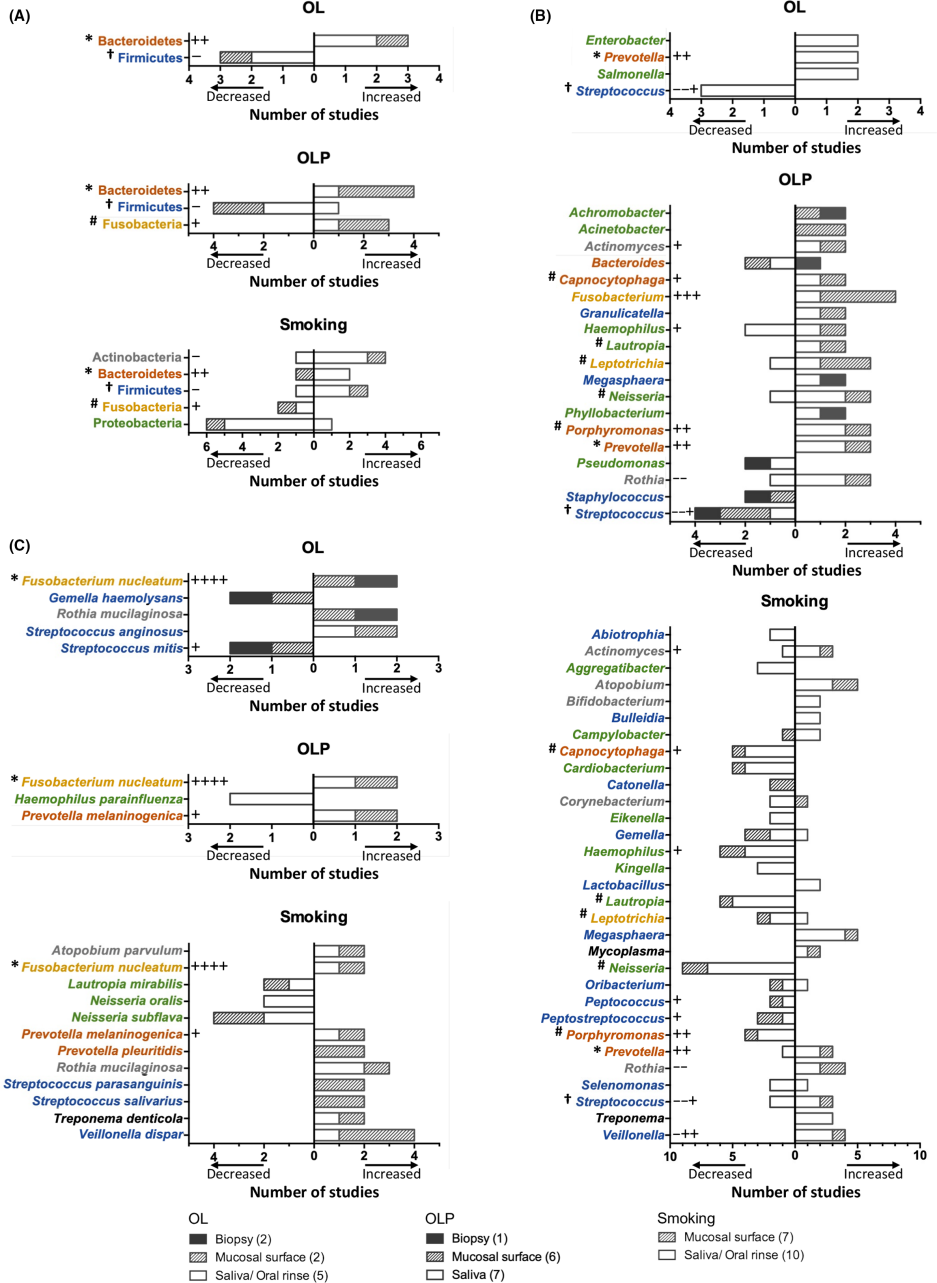
Significantly altered taxa repeatedly reported in OPMD‐associated studies on oral bacteriota. List of phyla (A), genera (B), and species (C) significantly increased or decreased in the indicated number of studies (only taxa reported in two or more studies are shown). *All three OPMDs have same direction with OSCC; ^†^Different between OL and Smoking; ^#^Different between OLP and Smoking; +, increased in OSCC; −, decreased in OSCC.

Of the nine OL‐associated oral bacteriota studies, seven reported α‐ and β‐diversities, referring to the diversities within and between the samples, respectively. The α‐diversities did not prove to be significantly different in four studies, whereas the significant differences found in the other three studies were rather inconsistent. Five studies reported significantly different bacterial β‐diversity associated with OL lesions, but two of four studies using saliva or oral rinse reported no significant difference. This suggests that changes in the overall structure and composition of oral bacteriome associated with OL are more evident at the local lesion than in the whole mouth. Regarding the taxonomic signatures at the phylum, genus, and species levels, four, four, and five studies, respectively, reported significant changes compared to health. There are common findings across the studies, regardless of the type of samples analyzed (Figure 2). For example, the depletion of phylum Firmicutes and the genus *Streptococcus* within that phylum were observed in three of the four studies. Similarly, the enrichment of phylum Bacteroidetes and genus *Prevotella*, a member of Bacteroidetes, was repeatedly observed. At the species level, increases in *F. nucleatum*, *Rothia mucilaginosa*, and *Streptococcus anginosus* and decreases in *Gemella haemolysans* and *Streptococcus mitis* were reported in two studies. An enrichment of phylum Bacteroidetes, genus *Prevotella*, and *F. nucleatum* species, along with the depletion of phylum Firmicutes, genus *Streptococcus*, and *S. mitis*, were observed in both OL and OSCC (Figure 2).

Regarding the OLP studies, all 13 reported α‐diversities. Seven studies (mainly in saliva) reported no significant differences, whereas five studies reported an increased Shannon diversity (three in mucosal swabs and two in saliva) and two studies reported a decreased richness in mucosal swabs. Of the 11 OLP studies that analyzed β‐diversity, 6 reported a difference in OLP, particularly in biopsy and mucosal swabs. In terms of taxon comparison, 9, 11, and 7 studies reported significant changes at the phylum, genus, and species levels, respectively. Increases in phylum Bacteroidetes and included genera, such as *Porphyromonas*, *Prevotella*, and *Capnocytophaga*, were repeatedly found for both OL and OLP, in four studies. While four studies reported the depletion of Firmicutes, two studies reported their enrichment in OLP. This discrepancy may be attributed to the decreases in genera *Streptococcus* and *Staphylococcus*, with concomitant increases in genera *Granulicatella* and *Megasphaera*, all of which belong to Firmicutes. Increases in phylum Fusobacteria, genus *Fusobacterium*, and species *F. nucleatum* were also repeatedly observed. Several genera, such as *Bacteroides*, *Haemophilus*, *Leptotrichia*, *Neisseria*, and *Rothia*, presented conflicting results across the studies (Figure 2). Such variations were often observed within the same type of samples; thus, the discrepancy is likely to be due to different distribution of species within the genera. Only one study analyzed bacteriota within the biopsy tissues and reported the dual enrichment of *Escherichia–Shigella* genera compared with healthy controls. The enrichments of genus *Escherichia* and species *E. coli* have also been reported within OLP tissues compared with OLP mucosal surface.^62^ Enrichment of phyla Bacteroidetes and Fusobacteria, genera *Prevotella*, *Fusobacterium*, and *Porphyromonas*, and species *F. nucleatum* and *Prevotella melaninogenica*, along with depletion of Firmicutes and *Streptococcus*, were observed in both OLP and OSCC (Figure 2).

## 9 SMOKING‐ASSOCIATED BACTERIAL DYSBIOSIS

Smoking is a strong etiologic factor for OSCC, OL, and OE^63^ but not for PVL, OSF, and OLP.^64^, ^65^, ^66^ Smoking‐induced oral microbial dysbiosis has been well documented and is associated with OSCC or OPMD. A total of 14 studies that compared bacteriota in the oral mucosa, saliva, or oral rinse of smokers and nonsmokers were collated (Table 4). Of those studies, 10 and 13 reported α‐ and β‐diversities, respectively. Only 3 of 10 studies reported significant differences in α‐diversity, which were inconsistent: increased Shannon diversity was observed in one study using saliva, but decreased Shannon diversity was observed in two studies using saliva and mucosal swab samples. However, 9 of 13 studies reported a significant difference in β‐diversity by smoking, which was more prevalent in saliva/oral rinse samples, than in mucosal swab samples. By the comparison of each taxon, 7, 12, and 6 studies reported significant changes at the phylum, genus, and species levels, respectively. In the smoker's oral cavity, increases in phyla Actinobacteria, Bacteroidetes, and Firmicutes and decreases in phyla Proteobacteria and Fusobacteria were repeatedly observed. Among the significantly changed genera belonging to Proteobacteria, the abundances of *Aggregatibacter*, *Cardiobacterium*, *Eikenella*, *Haemophilus*, *Kingella*, *Lautropia*, and *Neisseria* were decreased, whereas *Campylobacter* increased. A decrease in *Neisseria* was particularly found in nine studies, marked by decreases in *N. oralis* and *N. subflava* at species level. Among the altered genera that belong to Actinobacteria, *Actinomyces*, *Atopobium*, *Bifidobacterium*, and *Rothia* were increased, but *Corynebacterium* was decreased. Similarly, among the altered genera that belong to Firmicutes, *Bulleidia*, *Lactobacillus*, *Megasphaera*, *Streptococcus*, and *Veillonella* were increased, but *Abiotrophia*, *Catonella*, *Gemella*, *Oribacterium*, *Peptococcus*, *Peptostreptococcus*, and *Selemonas* were decreased. Interestingly, enrichment of *F. nucleatum* was reported in two studies, despite the depletion of phylum Fusobacteria (Figure 2).

**TABLE 4:** Characteristics of microbiome studies on smoking.

Authors	Sample	Group	Country	Method	Statistics	α‐diversity	β‐diversity	Comments
Wu et al., 2016^102^	Oral rinse	521 NeS 571 FS 112 CS	USA	16S rRNA V3‐V4	*q* < 0.05	–	Different	Consideration of smoking dose, duration, age, sex, race, and BMI Adjustment for age and sex
Yu et al., 2017^103^	Buccal swab	20 NoS 22 CS	USA	16S rRNA V3‐V4	*q* < 0.05	N.S	N.S	Consideration of smoking dose, duration, age, sex, race, drinking, and periodontal status Exclusion: antibiotics treatment or professional dental cleaning within 3 months, periodontal disease, cancer, and losing >1 tooth
	Tongue swab	18 NoS 19 CS				N.S	N.S	
	Saliva	16 NoS 18 CS				N.S	N.S	
Rodríguez‐Rabassa et al., 2018^104^	Saliva	16 NeS 18 CS	Puerto Rico	16S rRNA V3‐V4	*q* < 0.05	‐	Different	Consideration of smoking dose, duration, age, sex, race, education, drinking, and depression symptoms Exclusion: antibiotics therapy within the year
Karabudak et al., 2019^105^	Buccal swab	20 NoS 20 S	Turkey	16S rRNA V2, V3, V4, V6‐V7, V8, V9	*q* < 0.05	N.S	N.S	Consideration of smoking dose, age, sex, and BMI Exclusion: antibiotics within 3 months, respiratory infections, oral aphthous lesions, and dental problems
Al‐Zyoud et al., 2019^106^	Saliva	51 NoS 49 S	Jordan	16S rRNA V3‐V4	*q* < 0.05	↓	Different	Consideration of smoking dose, duration, age, sex, race, education, and oral hygiene Exclusion: antibiotics therapy within 3 months and history of chronic oral diseases
Lin et al., 2019^107^	Saliva	30 NoS 30 S	USA	16S rRNA V4	*q* < 0.05	‐	Different	Consideration of nicotine dependence Consideration of and adjustment for age, sex, drinking, and marijuana smoking Exclusion: nicotine use treatment, serious medical or psychiatric conditions within 6 months, brain‐related medical problems, bipolar or psychotic disorders, illicit drug use, and currently taking insulin or oral hypoglycemic medication
Yang et al., 2019^108^	Oral rinse	547 NeS 477 FS 592 CS	USA	16S rRNA V4	*q* < 0.05	‐	Different	Consideration of and adjustment for age, sex, race, BMI, drinking, total energy intake, oral health status, and disease status Exclusion: antibiotics usage during the year
Beghini et al., 2019^109^	Oral rinse	43 NeS 43 FS 83 CS	USA	16S rRNA V4	*q* < 0.05	N.S	Different	Consideration of smoking dose and serum cotinine level Consideration of and adjustment for age, sex, race, physical activity, education, diabetes status, and self‐reported gum disease
Al Bataineh et al., 2020^110^	Buccal swab	50 NoS 55 S	UAE	Metagenomics	*q* < 0.05	N.S	Different	Consideration of smoking duration and nicotine dependence Exclusion: antibiotic or probiotic usage within 3 months and pre‐existing respiratory illness
Sato et al., 2020^111^	Tongue swab	384 + 106 NeS 129 + 41 FS 144 + 40 CS	Japan	16S rRNA V3‐V4	*q* < 0.05	↓	Different	Consideration of smoking dose, duration, and age started smoking Consideration of and adjustment for age, sex, BMI, drinking, number of teeth, caries, and periodontal status Exclusion: age <20 or >90, hypertension, diabetes, probiotics, antimicrobial or steroids usage, current or former drinkers, former smokers, edentulous, and kidney problems
Sato et al., 2020^112^	Tongue swab	234 NeS 52 CS	Japan	Metagenomics	*q* < 0.05	‐	Different	Consideration of smoking dose and duration Consideration of and adjustment for age, sex, BMI, teeth number, caries, and periodontal status Exclusion: age <20 or >90, hypertension, diabetes, antimicrobial or steroids usage, current or former drinkers, former smokers, edentulous, and kidney problems
Wirth et al., 2020^113^	Saliva	11 NoS 11 CS	Hungary	Metagenomics	*q* < 0.05	N.S	N.S	Consideration of smoking dose, duration, age, sex, missing teeth, and periodontal status Exclusion: chronic illness, antibiotics therapy within 6 months, and moderate or severe periodontitis
Jia et al., 2021^114^	Saliva	150 NeS 166 S (CS + FS)	China	16S rRNA V4	LDA > 2 & *q* < 0.05	↑	N.S	Consideration of age, sex, and education
Suzuki et al., 2022^115^	Tongue cleaner	31 NoS 17 S	Japan	16S rRNA V3‐V4	*p* < 0.05	N.S	‐	Consideration of age, sex, and periodontal status; no antibiotics usage within 3 months
	Saliva	31 NoS 16 S				N.S	‐	

Abbreviations: CS, current‐smokers; FS, former‐smokers; LDA, linear discriminant analysis; N.S, not significant; NeS, never‐smokers; NoS, nonsmokers; S, smokers.

Smoking‐ and OSCC‐associated bacteriota share an enrichment of phylum Bacteroidetes, genera *Prevotella* and *Campylobacter*, and species *F. nucleatum* and *P. melaninogenica*. Among the taxa altered in OL, 1/2 phyla, 1/4 genera, and 2/5 species are common with those altered in smokers, whereas among the taxa altered in OLP, 1/3 phyla, 4/19 genera, and 2/3 species are common with those altered in smokers. Yet, 2/3 phyla and 6/19 genera are changed in the opposite direction from those altered in smokers. Hence, there are more opposing than common changes in the oral bacteriota between OLP patients and smokers. This underscores the role of the oral bacteria in the development of OLP, given that the prevalence of this oral pathology is significantly lower in smokers than in nonsmokers.^66^ Finally, the enrichment of Bacteroidetes, *Prevotella*, and *F. nucleatum* is a common feature shared among smoking‐, OLP‐, OL‐, and OSCC‐associated oral bacteriota, sparking additional interest in these taxa.

## 10 THE HEALTHY ORAL MYCOBIOME

Most of the focus on microbial dysbiosis has been placed on the bacteriota, frequently referred to as the “microbiome” component. In fact, “microbiome” has been often interchangeably used to refer entire microbiota, hence creating occasionally confusion for the reader. Although fungi are a minor component of the overall microbiota, they are bound to interfere significantly with dysbiosis and subsequently cause tissue damage to oral mucosa. Therefore, it is important to be aware of the fungal component of the microbiota or “mycobiome” in health, which will enable us to evaluate whether mycobiome dysbiosis could be associated with mucosal pathologies.

Some fungi are integrated as a part of human microbiota and are found in various human niches. However, they are a very small part of the entire microbial community compared to the bacterial component of the microbiome. Compared to the bacteriome, the oral mycobiome is found to constitute less than 0.06% and 0.0001% of the microbial community in saliva and dental plaque, respectively.^67^ The presence of low‐abundance fungi in the oral mycobiome does not necessarily indicate dysbiosis, as they could be environmentally acquired and transient occupants. Fungi commonly found in indoor and outdoor environments, as well as in food, can be continuously acquired by the host. Some fungi may not be able to grow in the oral cavity due to microenvironmental factors (e.g., suboptimal temperature) or competition for nutrients with bacteria. Knowledge of which fungi can replicate in the oral environment is important to avoid meaningless interpretations of mycobiome datasets.^68^, ^69^ Additionally, the sensitivity and low detection levels of some fungi raise the question of whether their presence is a transient finding or whether they are indeed commensals or pathobionts.

With the advent of sequencing techniques, bacterial taxa from human samples have been identified through amplification and sequencing of bacterial 16S rRNA genes. The fungal taxa have been explored using sequencing 18S rRNA and internal transcribed spacer (ITS) between fungal rRNA genes.^70^ Hence, “mycobiome” research has been largely based on these amplicon‐based sequencings prior to the arrival of metagenomics studies. In order to understand the role of the mycobiome in oral mucosal diseases, one must first decipher its symbiotic moiety that characterizes a healthy state. The first such study used oral rinse samples of healthy individuals of various ethnicities who consumed “western” diet.^71^ Sequencing of the mycobiome revealed 74 culturable and 11 nonculturable fungal genera, with between 9 to 23 culturable species present in each individual. The study defined that the healthy core (basal) mycobiome includes the fungal genera *Alternaria*, *Aspergillus*, *Aureobasidium*, *Candida*, *Cladosporium*, *Cryptococcus*, *Dothioraceae*, *Eurotium*, *Fusarium*, *Glomus*, *Saccharomyces*, *Saccharomycetales*, and *Teratosphaeria*. A following study identified additional fungal genera in the salivary oral mycobiome, including *Malassezia*, *Irpex*, *Cytospora/Valsa*, *Lenzites/Trametes*, and *Sporobolomyces/Sporidiobolus*, that may also qualify as members of the healthy core mycobiome.^72^

## 11 ORAL MYCOBIOME IN ORAL DYSPLASIA

Ever since these seminal studies on the healthy core mycobiome, several other studies have also delved into the dysbiotic mycobiome of various patient cohorts.^68^ A high variability is reported in the healthy mycobiome community, with *Malassezia* spp. being the most common shared predominant taxa. Yet, patients with active mucosal infections are dominantly colonized by *Candida* spp.^73^ Analysis of mycobiome of supragingival biofilms has revealed *Candida*, *Malassezia*, *Cryptococcus*, and *Trichoderma* spp. as the most prevalent/abundant taxa.^67^ The salivary mycobiome of cohort of 59 patients diagnosed with nonoral solid tumors, scheduled to receive chemotherapy, was studied using ITS1 amplicon sequencing.^74^ Two distinct fungal communities or “mycotypes” were identified, with *Candida* and *Malassezia* spp., respectively. The *Candida* spp. mycotype was associated with a lower microbial diversity and positively correlated with cancer occurrence, steroid use, smoking, and dental caries. Aciduric species were enriched in the *Candida* spp. mycotype, while inflammophilic species were enriched in the *Malassezia* spp. mycotype. This denotes that dysbiosis of the oral mycobiome is inter‐related to the dysbiosis of oral bacteriome in the pathologies of the oral cavity.

Hitherto only limited number of studies have examined the role of the oral mycobiome in oral mucosal diseases, due to the low prevalence of individual fungal species and lack of reference genomes. The first study to address the role of the oral mycobiome on OSCC indicated a dysbiotic mycobiome, which was characterized by reduced species richness and diversity.^32^ The relative abundance of *C. albicans* and *Candida etchellsii* were higher in OSCC, whereas noncancerous tissues fostered fungal taxa such as *Malassezia*, *Aspergillus*, *Alternaria*, *Cladosporium*, and *Hanseniaspora*, which are also known to produce anticancer compounds. In highly diverse microbial communities, the abundance of individual fungal and bacterial species is kept under control. However, when microbial diversity is reduced, such as in the case of antibiotic use, certain species become more abundant.^75^ Hence, during co‐infection, fungi and bacteria can promote each other's virulence, for instance, by aiding in tissue penetration through joint mechanisms.

## 12 THE PARTICULAR ROLE OF *CANDIDA* SPP. IN ORAL DYSPLASIA

When a *Candida* spp. colonizes the oral tissues, it can produce various virulence factors that may contribute to the development of oral dysplasia and cancer. One of the key mechanisms for the induction of oral dysplasia is through the production of carcinogenic metabolites. Studies have shown that *Candida* spp. can produce a variety of carcinogenic metabolites, such as nitrosamines, acetaldehydes, and reactive oxygen species (ROS). These can damage the DNA of oral cells, thus causing mutations that promote oral dysplasia and cancer. Alcohol consumption is a known risk factor for the development of oral cancer. Acetaldehyde, a carcinogenic compound produced by the metabolism of ethanol by the enzyme alcohol dehydrogenase (ADH1), can induce DNA and protein adducts that interfere with normal DNA replication, activate proto‐oncogenes, and cause cell cycle disturbances and tumor development. Studies have shown that *Candida* species, including *C. albicans*, *C. tropicalis*, and *C. parapsilosis*, produce acetaldehyde from ethanol, which can promote carcinogenesis. *Candida* spp. also produce nitrosamines, another carcinogenic compound that can bind with DNA and cause miscoding or irregularities in its replication. Additionally, chronic inflammation inflicted by *Candida* spp. upon oral tissues can contribute to the development of dysplasia and cancer. It is, however, unclear whether *Candida*‐induced inflammation facilitates the establishment of a tumor‐promoting microenvironment or whether a cancerized field with immune dysfunction creates secondary susceptibility to fungal infections, as discussed earlier.

## 13 ORAL MYCOBIOME IN OTHER MUCOSAL DISEASES

Apart from OSSC, other oral mucosal diseases in which the oral mycobiome is implicated include atrophic glossitis, OLP, and recurrent aphthous stomatitis (RAS). *Candida albicans* is a highly prevalent fungus in atrophic glossitis patients and it is associated with chronic mucosal inflammation in this pathology. A pilot study on salivary microbial diversity found that *Lactobacillus* and *Saccharomycetales* spp. were the most prevalent genera in atrophic glossitis patients, but there is not enough evidence to establish a direct link to the development or progression of the disease. OLP affects approximately 0.5%–2% of the general adult population, with variations in clinical presentation, including reticular, atrophic, and ulcerative lesions. *Candida* spp. can be detected at rates of 37%–50% in OLP patients, whereas their oral mycobiome is complemented further by *Alternaria*, *Aspergillus*, *Cladosporium/Davidella*, *Saccharomyces*, *Phoma*, *and Malassezia* spp.^76^

## 14 LIMITATIONS AND FUTURE DIRECTIONS

Despite a wealth of research on the role of the oral microbiome on the neoplastic onset in mucosa, many unanswered questions remain. The levels of certain oral bacteria or fungi might serve as a clinical indicator for oral mucosal lesions or OSCC, yet more research is required for confirmation. Several studies have comparatively investigated the oral microbiota found in both malignant and nonmalignant mucosal samples. Nevertheless, epidemiological studies indicate temporal and spatial associations between oral microbiota and these mucosal lesions, thus hinting at a plausible involvement in the initiation or progression of the disease. For proper investigation of compositional shifts of the oral microbiome in oral cancer, it is necessary to control for differences between oral subsites and inter‐individual variations, since cancers tend to develop out of a field of genetically altered cells (i.e. the concept of “field cancerization”). The oral microbiome is affected by diverse factors, including ethnicity, age, sex, oral disease, oral hygiene, smoking, alcohol consumption, and oral dryness. Many studies have not taken under consideration these parameters. Considering the age span of OSCC patients, screening for medications that can cause oral dryness or measurement of salivary flow rates is important, but most studies did not evaluate these. In addition, many studies tend to have differences in confounding factors between the study groups, and only some adjusted for such, whereas others were of too small sample sizes to allow for adjustment. Methodological variations in sample collection processes (stimulated/unstimulated saliva, surface swabs, or biopsies) and control sample types (tumor‐adjacent, clinically normal, contralateral to tumor, or benign lesions) are also contributing to the diversification of the results. Overcoming these methodological obstacles in microbiome data interpretation would require re‐analysis of raw datasets deposited in public databases, using a common bioinformatic pipeline or platform, rather than driving conclusions based on data analysis from individual studies. Hence, there is a need for central deposition of the sequencing data and meta‐data from various studies that would facilitate a more holistic re‐analysis and interpretation. There is also need for longitudinal follow‐up studies in order to confirm and validate the role of specific microbiota as risk indicators for developing a certain mucosal lesion or cancer, that is, independent of the commonly accepted and established risk factors.

Finally, it is important to note that some bacteria and fungi, or their metabolic compounds, such as antibiotics, bacteriocins, nonribosomal peptides, polyketides, or toxins, can exhibit anticancer cell bioactivity.^77^, ^78^ Putative mechanisms of action include apoptosis, necrosis, reduced angiogenesis, inhibition of protein translation, and inhibition of essential signaling pathways in cancer cells.^78^ Health‐associated species, such as *Streptococcus mitis*, *Rothia mucilaginosa*, *Neisseria flavescens*, *Haemophilus parainfluenzae*, *Lautropia mirabilis*, and *Veillonella parvula*, have been shown to inhibit proliferation of OSCC cells.^79^ Harnessing the anticancer properties of microbial agents may offer valuable alternative therapeutic options for cancer, particularly in addressing resistance to chemotherapeutic agents and their nonspecific toxic side effects.

## CONFLICT OF INTEREST STATEMENT

The authors declare no conflicts of interest.

## Data Availability

Data sharing is not applicable, since no new data were generated in this review work.
